# Worthy of Imitation

**Published:** 1841-09

**Authors:** 


### WORTHY OF IMITATION.

A young physician writes us as follows: "Early in the morning I study Latin—in the afternoon medical subjects—at night practise composition. When I am sufficiently advanced in the Latin, I wish in the morning to take up another language. Which do you think would be more profitable, Greek or French?" How many young physicians of the West are engaged in the laudable and systematic effort to supply the deficiencies of early education, and at the same time extend their professional knowledge? We fear the number is very small; and still nothing would be found more delightful than the course of this young gentleman. Early in the morning, physicians are not, in general, occupied, nor have they, commonly, much to do after dinner, and after dark. The forenoon and the evening are the chief business periods. If every young physician would devote himself to special studies, at special times of the day and night, he would not only form habits of the most beneficial kind, but, in a few years, would make acquisitions in literature and science, of which at the beginning he might not entertain the slightest anticipation. By passing the first years of their career (if that indolent existence which has no progression can be called career) without methodized courses of study, they stamp their characters with an indelible inferiority. Nothing is more common than to meet with physicians, in middle or more advanced life, who have made important observations, which, from illiteracy and inexperience in writing, they are unable to communicate to their brethren; or if they attempt it, their communications bring disgrace upon them, if not re-written, or at least corrected by the editor. Alas! for the poor editor. He is obliged to make bricks without straw. Much of what reaches him has to be committed to the flames to be refined—a part flares up, like straw or any other light matter—a part is too soggy to burn. Copying, transposing, abridging, inverting, rotroverting, decomposing and recomposing, are the labors which another class impose upon him. The third and last, *pars minor*, may only require new punctuation. It is quite impossible to express the joyousness of heart, inspired by one of *these* compositions. He feels an emotion of gratitude, from the moment he begins to discover, that bilious is not ll'd—apoplexy, pp'd—and camphor not introduced with a k: that symptoms are not followed by the verb singular, and that the phenomenon, of giving phenomena a plural verb, is actually before his ravished eyes. All such, he prints *verbatim et literatim* if not *punctatim*, and ever afterwards regards their authors, as his friends and benefactors. D.

